# Statistical methods for estimating the protective effects of immune markers using test-negative designs

**DOI:** 10.1093/aje/kwaf280

**Published:** 2025-12-29

**Authors:** Casey E Middleton, Daniel B Larremore

**Affiliations:** Department of Computer Science, University of Colorado Boulder, Boulder, CO, United States; BioFrontiers Institute, University of Colorado Boulder, Boulder, CO, United States; Department of Computer Science, University of Colorado Boulder, Boulder, CO, United States; BioFrontiers Institute, University of Colorado Boulder, Boulder, CO, United States; Santa Fe Institute, Santa Fe, NM, United States

**Keywords:** test-negative design, correlates of protection, logistic regression

## Abstract

While widely used to study vaccine effectiveness, test-negative designs (TNDs) also provide a platform for identifying and quantifying immunological correlates of protection against disease. A key component of such studies is the protection function, the mathematical relationship between the value of an immunological assay and the relative risk of disease. This function is often estimated using logistic regression, comparing the odds of disease at a given assay value to the odds at assay value zero. Here, we show through mathematical analysis and simulation experiments that logistic regression, while common, fundamentally constrains the functional forms of protection that can be inferred from data in TNDs, potentially leading to overly simplistic estimates of the protection function. To address this limitation, we adapt and analyze a scaled logit model, originally developed for case–control data, as a flexible alternative that allows for greater flexibility in estimating protection functions from TND data. We demonstrate that this approach improves accuracy across a range of biologically plausible protection functions, highlight conditions under which it may fail, and provide practical guidance for researchers to adopt it as a new standard for TND studies evaluating correlates of protection.

## Introduction

Antibodies are a key component of the adaptive immune system, equipping the host to quickly recognize and respond to pathogen invasion. Antibodies have been identified as a correlate of protection against infection or severe disease for pathogens such as flu,^1^ SARS-CoV-2,^2-8^ Ebola virus,^9^^,^^10^ and HIV.^11^ While the concentration of antibodies and other immune molecules can be qualitatively linked to decreased risks, establishing the quantitative relationship between immune markers and protection from empirical data is more challenging.

Test-negative design (TND) studies provide cost-effective data to understand the risk reduction provided by potential correlates of protection, and have been widely used to empirically estimate vaccine effectiveness (VE).^12-17^ These TNDs recruit individuals who seek care for a particular symptom set, such as influenza-like illness (ILI) or fever, and all recruited individuals are tested for a pathogen of interest. Test outcomes and patient covariates are reported alongside vaccination status, and logistic regression is then used to estimate the association between vaccination and risk reduction.^12^^,^^13^

More recently, TNDs have been extended beyond VE to understand the risk reduction associated with scalar correlates of protection.^2^^,^^3^^,^^6^^,^^7^^,^^18^ These studies rely on the key assumption that the antibody titers measured at the time of testing and recruitment are representative of titers at time of exposure and infection. In other words, because TNDs recruit subjects at the time of clinical presentation, TND studies of correlates of protection assume that titers are measured prior to boosting from infection.

TNDs studying scalar correlates of protection have, to date, used logistic regression to estimate risk reductions.^2^^,^^6^^,^^7^^,^^18^ Logistic regression provides a powerful statistical tool to estimate the odds of a binary outcome, such as observable disease, given covariates. However, while a logistic regression model’s ability to estimate the effect of binary correlates of protection (eg, vaccination) in TNDs is well characterized,^12^^,^^13^ scalar correlates of protection are more complicated because any regression model must assume a mathematical relationship—that is, a particular functional form—between titer and protection. We call this relationship a *protection function*. While protection functions for antibodies are typically found to be sigmoidal in randomized controlled trials, animal challenge models, and meta-analyses,^19-21^ it is unclear whether typical logistic regression, which recovers plausible protection functions from other study designs, is able to do so for TND data in particular.

In this manuscript, we make progress on the problem by first showing which mathematical relationships may be well suited for inference using logistic regression in TND studies, and which may not. While the protective effects of antibodies are typically assumed to be sigmoidal in the literature,^19-21^ we demonstrate that standard logistic regression applied to TND data inherently limits the ability to accurately recover this functional form of protection. We then extend the scaled logit model introduced by Dunning^22^ to TND data, demonstrating its ability to recover a broader class of protection functions from TND studies. We explore the accuracy of the scaled logit model for inference of various protection functions, demonstrate its utility on empirical TND data, identify conditions under which it may fail, and provide practical guidance for researchers to adopt this model as a new standard for estimating the protective effects of immunological assays in TND studies.

## Methods

To evaluate the ability to accurately learn a protection function from data using statistical estimation methods, we developed a synthetic TND data testbed. Test-negative design studies recruit individuals who report a particular symptom or symptom set. Recruited individuals are tested for a pathogen of interest, with data reported on: (1) test result (positive or negative), (2) value of covariate(s) assumed to be associated with infection risk (ie, vaccination status or immunological assay), and (3) other variables that may impact risk (ie, age, sex). Our synthetic data testbed mirrors this reporting schema for scalar $\log$ antibody titers.

### Generating synthetic test-negative design data

We generated a synthetic TND data testbed by first specifying a protection function, $\varPhi (A)$ (see Supplement for function descriptions). To generate titer distributions for test-negative controls, we drew $A$ from a known distribution ($\mathit{\log}(A)\sim \log -\mathrm{uniform}\mathrm{\left(1,10\right)}$ in main text). To generate titer distributions for test-positive cases, we used a rejection sampling technique such that a candidate titer was drawn from the same distribution as controls, and was then rejected with probability $1-\varPhi (A)$, simulating the protective effects of antibodies. Samples were drawn to achieve a specified total sample size $N$ and a chosen case-to-control ratio $c$. Unless otherwise specified, we assumed a large sample size ($N{=\mathrm{10}}^5$) and perfect diagnostic sensitivity and specificity when assigning infected status.

### Inferring the protection function from data

Given a set of $\log$ antibody titers and disease outcomes, the protection function was estimated in two steps. First, a statistical model was used to infer the risk of disease given antibody titers and other covariates. We utilized logistic regression as the typical statistical approach,^2^^,^^6^^,^^7^^,^^18^ and also demonstrated a scaled logit model alternative^22^ (see Supplement for model fitting protocol). From this risk estimate, the protection function was computed using the odds ratio (OR) definition $1-\mathrm{OR}$, comparing the odds of testing positive with antibody titer $A$ against the baseline odds at $A=\mathrm{0}$.

## Results

### Standard logistic regression recovers only exponential protection functions from TND data

Test-negative designs (TNDs) are commonly used to estimate vaccine effectiveness (VE), defined as the reduction in risk of disease due to vaccination. In TNDs, VE is estimated via the adjusted odds ratio (OR) of disease among vaccinated vs unvaccinated individuals, that is, $VE=\mathrm{1}- OR$_._^12^^,^^13^ Logistic regression is a convenient statistical method for estimating this OR and is often employed in the analysis of TND data, primarily because it enables one to control for other relevant covariates $\theta$_._^12^^,^^14-16^^,^^23^ In a logistic regression model, the log-odds of disease given vaccination status and other covariates is given by,


(1) \begin{equation*} \mathrm{logit}\ p={\beta}_0+{\beta}_VV+{\beta}_1{\theta}_1+{\beta}_2{\theta}_2+\dots, \end{equation*}

where $p$ is the probability of testing positive given symptoms, $V$ is an indicator for vaccination status, and ${\theta}_i$ represent other covariates. The coefficient ${\beta}_V$ represents the change in log-odds of disease associated with vaccination, meaning the OR is $OR={e}^{\beta_V}$. Therefore, VE is defined as,


(2) \begin{equation*} VE=\mathrm{1}-{e}^{\beta_V}. \end{equation*}

When TNDs are used to evaluate scalar correlates of protection, such as $\log$ antibody titer, a typical approach is to replace binary vaccination status $V$ with a measured titer $A$ and proceed with typical logistic regression.^2^^,^^6^^,^^7^^,^^18^ Relative to a baseline risk when $A=\mathrm{0}$, this replacement yields a protection function $\varPhi$, which describes the fraction by which risk is reduced as a function of $A$,


(3) \begin{equation*} \varPhi (A\mathrm{)=1}-{e}^{\beta_AA}, \end{equation*}

where ${\beta}_A$ is the change in log-odds of risk given a 1-unit increase in $\log$ antibody titer.

Equation (3) provides the exact functional form of the induced protection function when logistic regression is used to learn from TND data. Consequently, logistic regression is well suited to recovering this particular functional relationship between titers $A$ and protection $\varPhi$. Hereafter, we refer to (3) as an exponential protection function.

**Figure 1:**
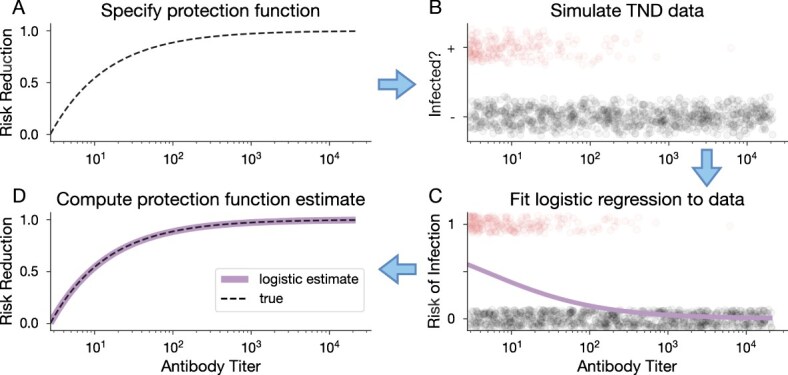
Logistic regression accurately estimates exponential protection functions. (A) First, we specify an exponential protection function using eqn (3) and some ${\beta}_A<\mathrm{0}$. (B) Next, we simulate test-negative design (TND) data using this protection function to produce antibody distributions for test-positive (+, red circles) and test-negative (-, gray circles) individuals (subset of data shown here). (C) We use logistic regression to estimate risk of infection from the simulated data. (D) Lastly, we compute the estimated protection function from the logistic regression model results (solid purple curve), which we compare to the true protection function (dashed black curve) that was used to generate the data, specified in step A.

To illustrate how logistic regression accurately recovers exponential protection functions, we utilize our synthetic TND data testbed. First, we specify the shape of an exponential protection function (eqn (3); Figure 1A) with some ${\beta}_A<\mathrm{0}$ and use this protection function to simulate infected and uninfected antibody titer distributions (Figure 1B). We use logistic regression to estimate disease risk from the simulated data (Figure 1C). This risk estimate is then used to compute the OR with a baseline of $A=\mathrm{0}$, to arrive at an estimated protection function (Figure 1D). This simulation illustrates what we have shown mathematically: using logistic regression to infer risk can recover the true protection function when that function is exponential. Statistically speaking, when the logistic regression model is correctly specified for the true protection function, estimation is consistent.

### Logistic regression is poorly suited to recover a sigmoidal protection function from TNDs

The fact that logistic regression implies a protection function defined by a single-parameter exponential (eqn (3)) raises an important question: what if the true protection function is not exponential? We illustrate this type of mismatch between model and data—a model misspecification problem—through a second simulation experiment that follows the same 4-step procedure illustrated above. However, in this experiment, we generate data according to a plausible (but nonexponential) alternative protection function, and then fit a logistic regression model to the data.

**Figure 2:**
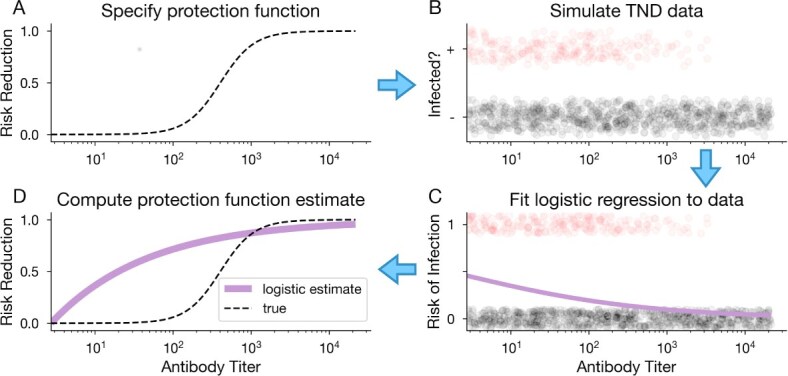
Logistic regression does not accurately estimate sigmoidal protection from TND data. (A) First, we specify a sigmoidal protection function using eqn (4) and some ${\beta}_0>\mathrm{0}$, ${\beta}_A<\mathrm{0}$. (B) Next, we simulate TND data using this protection function to produce antibody distributions for test-positive (+, red circles) and test-negative (-. gray circles) individuals (subset of data shown here). (C) We use logistic regression to estimate risk of infection from the simulated data. (D) Lastly, we compute the estimated protection function from the logistic regression model results (solid purple curve), which we compare to the true protection function (dashed black curve) that was used to generate the data, specified in step A.

Rather than an exponential protection function, we used a sigmoidal shape from which to simulate (Figure 2A), as is commonly found in randomized controlled trials, animal challenge models, and meta-analyses.^19-21^ The sigmoidal protection function is defined by


(4) \begin{equation*} \varPhi (A\mathrm{)=1}-\frac{1}{1+{e}^{-\left({\beta}_0+{\beta}_AA\right)}}, \end{equation*}

for some ${\beta}_0>\mathrm{0}$, ${\beta}_A<\mathrm{0}$. We simulated TND data using this sigmoidal protection function (Figure 2B), and fit a logistic regression model to the synthetic data (Figure 2C). The resulting estimated protection function (Figure 2D) is no longer accurate.

Logistic regression fails to recover the true sigmoidal protection function used to generate the data, as evidenced by the mismatch between the estimated and true curves (Figure 2D). These results are not unexpected: the logistic regression model is restricted to exponential protection functions in TNDs due to their particular reliance on the exposure OR (eqn (3)). Thus, logistic regression cannot recover other functional forms, even when applied to an ideal dataset. To infer a potentially nonexponential relationship between antibodies and protection, a different statistical approach is required.

### The scaled logit as an improved model for scalar correlates of protection in TND data

A few alternatives have been proposed for inferring the functional relationships for correlates of protection, which we briefly review. Zhang *et al*. show that rescaling model inputs such that $\mathrm{logit}p\sim \mathit{\ln}\mathrm{(1}-{X}^n)$ for titers $X\in \mathrm{\left[0,1\right]}$ can recover linear or polynomial protection functions,^24^ and further propose the use of semiparametric generalized additive models for flexible protection function inference. One might also consider modifying a typical logistic regression model, such that


(5) \begin{equation*} \mathrm{logitp}={\beta}_0+{\beta}_1A+{\beta}_2{A}^2+...+{\beta}_n{A}^n, \end{equation*}

an approach commonly employed when the relationship between the predictor and log-odds is nonlinear. Using our synthetic data testbed, we demonstrate that polynomial expansion cannot recover a sigmoidal protection function (Figure S1).

For case–control data, Dunning proposed a scaled logit model^22^ that defines the probability of observing a case as


(6) \begin{equation*} \Pr \left(\mathrm{disease}\right)=\lambda (1-\pi (A|\theta )). \end{equation*}

Here, $\lambda$ denotes the probability a fully susceptible individual develops disease, and $\pi \left(A\mid \theta \right)$ describes the probability that an individual with titer $A$ is protected, given model parameters $\theta$. If a logistic protection function is assumed for $\pi (A)$, the resulting disease risk is given by,


(7) \begin{equation*} \Pr \left(\mathrm{disease}\right)=\frac{\lambda }{1+{e}^{\beta_0+{\beta}_AA}}. \end{equation*}

The utility of this model has been demonstrated in meta-analyses^5^^,^^8^^,^^25^ and randomized controlled trials^26^^,^^27^ but never in TNDs.

We now extend the scaled logit model for use in TNDs by using eqn (7) to estimate the OR of protection at titer $A$ vs titer $A=\mathrm{0}$. This yields an estimated protection function of


(8) \begin{equation*} {\varPhi}_S(A\mathrm{)=1}-\frac{1-\lambda +{e}^{\beta_0}}{1-\lambda +{e}^{\beta_0+{\beta}_AA}}. \end{equation*}

Importantly, the scaled logit requires estimation of only 1 more parameter $\lambda$ than typical logistic regression, which in turn produces a protection function with 3 parameters ${\beta}_0$, ${\beta}_A$, and $\lambda$ (eqn (8)) instead of only 1 parameter given by logistic regression (eqn (3)). Consequently, we may expect protection functions resulting from the scaled logit model to be substantially more flexible in modeling correlates of protection.

**Figure 3:**
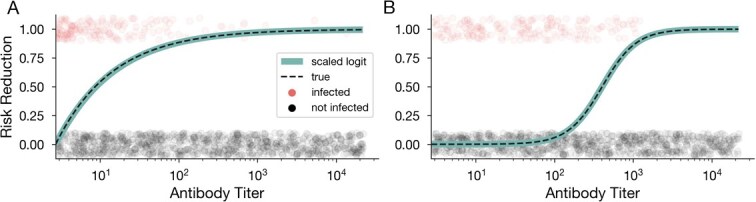
The scaled logit model can recover general antibody protection functions from TND data. Estimated protection function using the scaled logit model (solid green curve) trained on data generated from exponential (A) and sigmoidal (B) protection functions (dashed black curve). Circles show a subset of infected (red, vertical position 1) and uninfected (gray, vertical position 0) simulated individuals at various antibody titers used to train the model.

To demonstrate the flexibility of a scaled logit for TND studies, we simulated TND data under both exponential and sigmoidal protection functions (Figure 3A and B) and used the scaled logit model to estimate the protection function for each synthetic data set. We observe that the scaled logit model accurately fits both exponential and sigmoidal protection functions. These results demonstrate that the scaled logit model can recover not only exponential protection, which logistic regression can recover, but also sigmoidal protection functions, which logistic regression cannot.

To further explore the ability of these models to accurately recover a variety of protection function scenarios, we subjected both the scaled logit and logistic models to synthetic data generated from 2 additional protection functions, a shifted step function and a constant $\varPhi (A\mathrm{)=0}$ null result test. As with the sigmoidal protection function, the scaled logit model accurately estimated step-function protection, while logistic regression did not (Figure S2A). Both models accurately fit the null protection function $\varPhi (A\mathrm{)=0}$, confirming their ability to correctly find a true null result (Figure S2D). In further tests, we restricted the range of antibody titers such that few individuals had high or low titers, to challenge the scaled logit model’s ability to recover protection functions from less optimal data, but its high accuracy was unaffected (Figure S3).

These results demonstrate that introducing a single additional parameter in a scaled logit model enables inference of a broader range of functional forms from TND data, which standard logistic regression cannot capture. Establishing that the scaled logit model can accurately recover either sigmoidal or exponential protection functions from synthetic data indicates that we may be able to recover either functional form from real data, which we now turn to.

### The scaled logit model estimates sigmoidal curves from empirical TND data

Our synthetic data experiments demonstrate that the scaled logit model can recover a sigmoidal protection curve in TND studies, while logistic regression cannot. To understand how these findings extend to empirical data, we applied the same inference pipeline to published TND data that associates antispike antibody titers with SARS-CoV-2 infection, as previously described.^2^ This study enrolled 2300 patients between March 22, 2021, and August 17, 2022, who presented with undifferentiated acute febrile syndromes across 2 hospitals in the Dominican Republic. Nasopharyngeal swabs and sera from each patient were tested for SARS-CoV-2 infection using real-time PCR (rtPCR) and for total antispike antibodies with the Elecsys platform (Roche Diagnostics, Indianapolis, IN, USA). Positive samples with cycle threshold values less than 25 were sequenced using Oxford Nanopore or Illumina platforms (*N* = 216). These variant annotations allow inference of variant-specific protection functions by comparing variant-positive cases to a random sample of negative controls.

**Figure 4:**
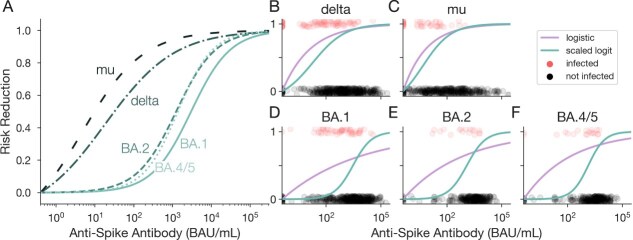
The scaled logit model allows for inference of a sigmoidal protection function from empirical TND data. Estimated protection function using the scaled logit model (green curves) and logistic regression (purple curves) fit to empirical TND data for SARS-CoV-2, stratified by variant of concern for (B) delta, (C) mu, (D) BA.1, (E) BA.2, and (F) BA.4/5. Circles show infected (red, vertical position 1) and uninfected (gray, vertical position 0) sampled individuals for each variant. Abbreviation: BAU, binding antibody units.

We first estimate 5 variant-specific protection functions using the scaled logit model stratified by variant (Figure 4A). Our results show that variants that emerged earlier, ie, delta and mu, are characterized by protection functions that increase rapidly to provide high levels of protection even at low titers. We estimate 50% risk reduction when antispike antibody titers reach ${10}^{1.51}$ and ${10}^{1.00}$ binding antibody units (BAU)/mL for delta and mu, respectively. The estimated protection functions for the omicron sublineages BA.1, BA.2, and BA.4/5 are more sigmoidal in shape, estimating 50% risk reduction at titer values of ${10}^{3.48}$, ${10}^{3.10}$, and ${10}^{3.14}$ BAU/mL, respectively. We note that stratifying by variant results in relatively small sample sizes and few positive cases, especially for BA.2 and BA.4/5 (17 and 19 cases, respectively).

Next, we compare the protection function estimated by the scaled logit model to that estimated by typical logistic regression, stratified by variant (Figure 4B–F). We observe a lower corrected Akaike information criterion (AICc) using the scaled logit model for 4 of 5 variants, with nearly equal scores for the mu variant, suggesting that the scaled logit model more accurately describes the empirical data (Table S1). For all variants, logistic regression underestimates the titer required to achieve 50% risk reduction when compared to the scaled logit model estimates. This underestimation is substantial for the omicron sublineages (BA.1, BA.2, BA.4/5), whose sigmoidal scaled logit protection curves deviate more drastically from the logistic estimates than the delta and mu variants. Given that the scaled logit model can reliably estimate either exponential or sigmoidal protection functions (Figure 3), the sigmoidal estimates observed across SARS-CoV-2 variants indicate that the true underlying protection mechanisms likely more closely resemble sigmoidal than exponential curves. These empirical results demonstrate that using standard logistic regression underestimates the amount of antibodies needed for 50% protection.

### Scaled logit model limitations

Test-negative designs studying correlates of protection vary widely in sample size, from a few hundred^6^^,^^18^ to several thousand.^2^^,^^7^ Like any model, the ability of the scaled logit and logistic regression to recover an underlying phenomenon is constrained for small sample sizes (explored in Figures S4-S6). While the addition of a scaling constant $\lambda$ allows for more flexible protection function estimates, ${\varPhi}_S$ still approaches 1 as $A\to \infty$ (eqn (8)) for typical monotonically decreasing risk parameterization (ie, ${\beta}_0<\mathrm{0}$, ${\beta}_A>\mathrm{0}$) at sufficient sample sizes. Because of this, the model may be ill suited to recover scenarios where protection is imperfect, or where near-perfect protection exists but is simply not represented in the sampled controls.

**Figure 5:**
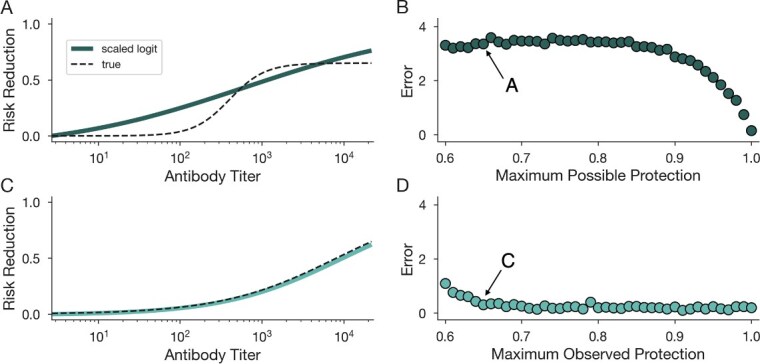
Scaled logit model has reduced accuracy if antibodies do not provide perfect protection. The scaled logit model is fit to TND data generated from a sigmoidal protection function which does not saturate to 1 (A, B) and when individuals with near-perfectly protective antibody titers are not sampled among controls (C, D). (A, C) Predicted protection functions from the scaled logit model (solid green curves) vs true protection (dashed black curves) for a maximum protection of 0.65. (B, D) Prediction error (Euclidean distance between predicted and true protection) under varying maximum protection levels.

We conducted two numerical experiments, each of which explores a different scenario in which the scaled logit may have poor accuracy. In the first experiment, we asked what happens if the protection function does not saturate to 1—that is, to perfect protection—as antibody titers grow large. We simulated TND data under a sigmoidal protection function that saturates at 65% (Figure 5A). We then estimated the protection function using the scaled logit model and computed error as the discrete ${\ell}_2$ norm of the difference between the best-fit model and the true protection function. The best-fit protection function overestimates protection at both low and high antibody titer, and approaches 1 as titer values increase. Varying the saturation point, or maximum possible protection, we observe relatively stable error estimates until the maximum possible protection surpasses 90%, at which point error approaches 0 (Figure 5B).

In the second experiment, we asked what happens if individuals with near-perfectly protective antibody titers are simply not sampled among the controls. To simulate this, we generated TND data where the maximum sampled antibody titer is ${A}_{\mathrm{max}}$, but risk reduction is perfect only for values *A ≫ A*_max_ (Figure 5C). For this scenario, we observe an estimated protection function that nearly perfectly estimates the true protection function. Varying the maximum observed protection shows that error is low and nearly constant across scenarios (Figure 5D).

**Figure 6:**
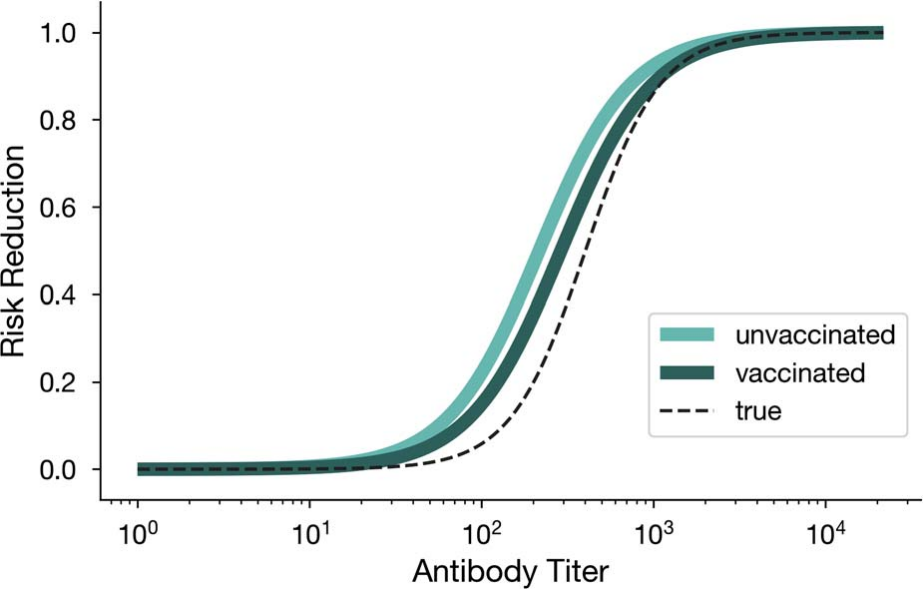
Unobserved correlates of protection lead to collider bias in protection function estimation. Estimated protection function using the scaled logit model (solid green curves) for vaccinated and unvaccinated individuals, when prior infection acts as an unobserved collider variable for risk reduction. Data is generated using a sigmoidal true protection (dashed black curve).

Finally, we explored a complication that arises in empirical data: observed titers may result from vaccination, prior infection, or both. Because prior infection may elicit a broader immune response than vaccination alone, measured titers may have a different relationship with protection in vaccinated vs unvaccinated people, making titer a collider variable. To explore this phenomenon in the context of protection functions, we generated synthetic data where measured antibodies could be generated by both vaccination and prior infection. All individuals benefit from antibody-associated protection via the protection function $\varPhi (A)$, but prior infection is treated as an unobserved latent variable uncorrelated with vaccination that elicits additional protection (see Supplement for details). Fitting the scaled logit model to titer data with vaccination status as a binary covariate leads to sigmoidal protection functions, illustrating the ability of the model to infer protection functions differentiated by vaccination status. As expected, these curves (1) overestimate the total protective effects of measured antibodies due to the presence of other unobserved sources of protection, and (2) illustrate the expected collider bias: at any fixed titer, vaccinated individuals appear less protected than unvaccinated (Figure 6).

## Discussion

Our results establish the scaled logit model as a more flexible alternative to typical logistic regression methods for estimating protection as a function of scalar immunological assays in TND studies. Using simulated data, we see that the scaled logit model can recover not only an exponential protection function, which logistic regression can recover, but also a sigmoidal protection function, which logistic regression fails to recover. These findings extend to real data, where we observe sigmoidal estimates of the protection function when characterized using the scaled logit model, compared to exponential estimates using logistic regression. These results lead us to recommend the adoption of the scaled logit model for modeling scalar correlates of protection from TND data.

Despite its increased flexibility, we also demonstrate that the scaled logit model may not accurately recover the true protection function for sample sizes below $1000$, and that model consistency deteriorates for sample sizes under ${10}^4$. Furthermore, we show that if protection does not saturate to 1, model accuracy may suffer. Alternative sigmoidal models with greater flexibility to capture protection functions that do not saturate to 1, exhibit asymmetry, or are characterized by extremely steep transitions have been previously explored,^28^ but may require model selection or prior knowledge of the expected shape of the protection curve. Alternative innovations using generalized additive models have also been proposed with promising results.^24^

Our methods are subject to a number of limitations. First, we simulate data under ideal sampling scenarios, exploring limitations of sample sizes and case-to-control ratio variations, but not sensitivity and specificity of diagnostic tests^16^^,^^29^ or noise in immunological assay measurements. Second, we do not consider the effects of demographic covariates, such as age and sex, or immunity covariates, such as time since last infection or other immunological assays.^30^ While these covariates may impact protection estimates, we posit that the scaled logit model can be used to control for their effects by including additional terms in eqn (7). Third, our exploration of protection functions is limited to functions that start at 0 when $A=\mathrm{0}$ and monotonically increase with increasing titer. These functions do not account for immune imprinting^31^ or antibody-dependent enhancement,^32^ which may lead to non-increasing protection functions. Lastly, our synthetic data testbed does not account for differences in care-seeking behavior or potential cross-protection between circulating pathogens, and assumes that protection is “leaky” instead of “all-or-nothing”, which can lead to differential depletion of susceptibles over the course of a season. These factors have been shown to potentially bias the protection estimates inferred using TNDs for VE studies^12-14^^,^^33^ and would likely also bias study results inferring correlates of protection.

Quantifying the relationship between assay value and risk reduction is an important task to understand the utility of immunological assays. Informative immunological assays may open up new data streams to model heterogeneous susceptibility in immune-experienced populations^34^^,^^35^ and vaccination strategies.^36^^,^^37^ Furthermore, understanding the strength of correlations between titer values and protection could be used to predict vaccine efficacy from titers alone instead of following a cohort in vaccine trials, reducing costs and increasing the speed at which trials may be performed. The scaled logit model, in combination with TNDs, may provide more accurate mathematical descriptions of how immunological measurements predict protection levels, opening the door for immunological assays to inform infectious disease modeling efforts, and establishing quantitative measurements of correlates of protection as substantially higher in value than binary alternatives.^38^

## Supplementary Material

Web_Material_kwaf280

## Data Availability

Implementations of scaled logit model fitting in R and Python are provided in Supplementary Materials. All code used for data simulation, model fitting, and reproducibility, as well as empirical data used in the study, can also be found at https://github.com/CaseyMiddleton/TNDforCOP.
