# Supplementary material for: Statistical methods for estimating the protective effects of immune markers using test-negative designs
Source: Am J Epidemiol. 2025 Dec 29;195(5):1357–64. doi: 10.1093/aje/kwaf280 (PMC13149038; doi:10.1093/aje/kwaf280)
Supplement: Web_Material_kwaf280 [file web_material_kwaf280.zip › Supplement.pdf]

# Statistical Methods for Estimating the Protective Effects of Immune Markers Using Test-Negative Designs

Casey E. Middleton[1,2] and Daniel B. Larremore[1,2,3]

[1] Department of Computer Science, University of Colorado Boulder, Boulder, CO, USA [2] BioFrontiers Institute, University of Colorado Boulder, Boulder, CO, USA [3] Santa Fe Institute, Santa Fe, NM, USA

**Included material:**

Figures S1 - S6

Table S1

Sample code for fitting the scaled logit model to data

# Supplement

## Protection functions

We considered four protection functions. These are the exponential, $\Phi(A) = 1 - e^{\beta_A A}$; the sigmoidal, $\Phi(A) = 1 - [1 + e^{-(\beta_0 + \beta_A A)}]^{-1}$; the step-function or threshold, $\Phi(A) = \Theta(A - A_0)$, where $\Theta$ is the Heaviside function; and the constant null model, $\Phi(A) = 0$.

## Fitting logistic regression models

The typical logistic model, that is logit $p = \beta_0 + \beta_1 A$, was fit to simulated TND data using `sklearn` (Python v3.8.11) without regularization (see Code Availability). Parameters were fit to log titer data, following typical procedure [1, 2, 6, 7]. The protection function was then computed using the odds ratio definition $1 - \text{OR}$, comparing the odds of testing positive with $\log$ antibody titer $A$ against the baseline odds of testing positive at $A = 0$.

## Fitting scaled logit models

The scaled logit model in Eq. (7) was fit to simulated TND data using maximum likelihood estimation (MLE). The likelihood of parameters given TND data is,

$$\mathcal{L}(\lambda, \beta_0, \beta_A; \{y_i, A_i\}) = \prod_i \left(\frac{\lambda}{1 + e^{(\beta_0 + \beta_A A_i)}}\right)^{y_i} \left(1 - \frac{\lambda}{1 + e^{(\beta_0 + \beta_A A_i)}}\right)^{1 - y_i}, \tag{S1}$$

where $A_i$ denotes individual antibody titers and $y_i \in \{0, 1\}$ denotes an observed non-infection or infection, respectively. We wish to find the parameters $(\lambda, \beta_0, \beta_1)$ which maximize this likelihood function, or equivalently the parameters that minimize the negative log-likelihood,

$$\mathcal{NLL}(\lambda, \beta_0, \beta_1; \{y_i, A_i\}) = \sum_i \left[-y_i \log\left(\frac{\lambda}{1 + e^{(\beta_0 + \beta_A A_i)}}\right) - (1 - y_i) \log\left(1 - \frac{\lambda}{1 + e^{(\beta_0 + \beta_A A_i)}}\right)\right]. \tag{S2}$$

This negative log likeilhood was fit to TND data using `scipy.minimize` (Python v3.8.1), implemented with the Nelder-Mead minimization algorithm [39] and options to increase the number of iterations before convergence. To prevent overfitting of small sample sizes, which results in a large $\beta_A$ slope parameter, we include regularization on the term $0.1 \times {\beta_A}^2$. Both Python and R implementations are provided at the end of Supplementary Materials.

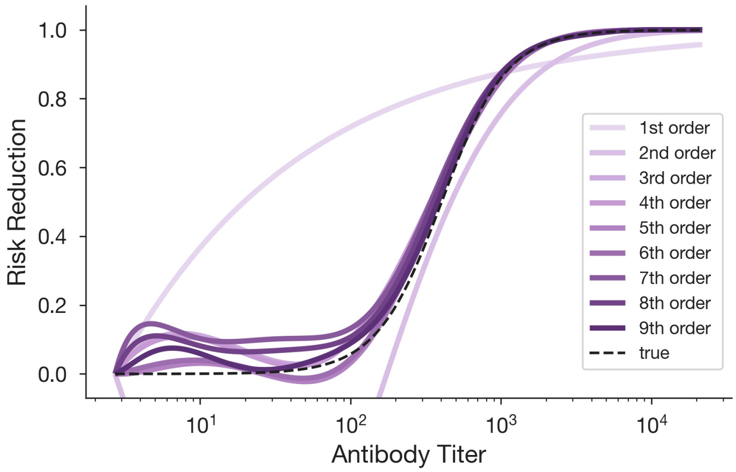


Figure S1: **Adding polynomial terms to logistic regression cannot recover a true sigmoidal protection function.** Protection function (purple) estimated using logistic regression with higher order polynomial input terms (as labeled). Data was generated using a sigmoidal protection function (dashed black curve).

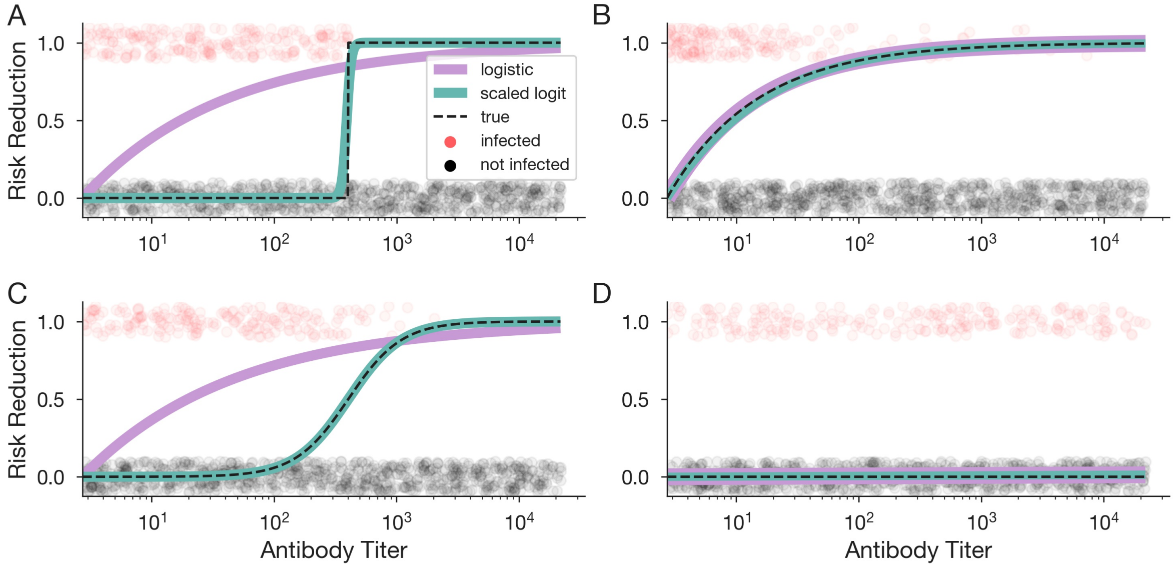


Figure S2: **The scaled logit model can recover more general antibody protection functions, while logistic regression is limited in its scope.** Estimated protection function using the scaled logit model (green curve) trained on data generated using a threshold (A), exponential (B), sigmoidal (C), and flat (D) protection functions (dashed black curve). Circles show a subset of infected (red) and uninfected (gray) simulated individuals at various antibody titers used to train the model.

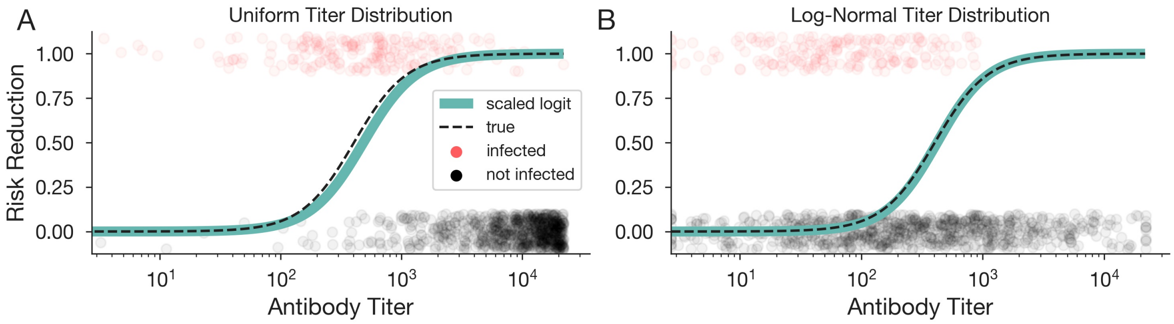


Figure S3: **The scaled logit model can recover more general antibody protection functions, even under different titer distributions.** Estimated protection function using the scaled logit model (green curve) trained on data generated using exponential (A) and sigmoidal (B) protection functions (dashed black curve) for uniformly (A) and normally (B) distributed titers. Circles show a subset of infected (red) and uninfected (gray) simulated individuals at various antibody titers used to train the model.

| | **Logistic Regression** | **Scaled Logit** |
|---|---|---|
| delta | 424.3 | 422.3 |
| mu | 247.1 | 247.9 |
| BA.1 | 412.1 | 326.3 |
| BA.2 | 144.1 | 129.9 |
| BA.4/5 | 153.2 | 139.0 |

Table S1: **Small-sample corrected Akaike information criterion (AICc) for logistic regression and scaled logit models, stratified by variant.** Green highlights denote the model which best explains the data for each variant.

## Scaled logit model accuracy depends on sample size

Studies vary widely in the total number of individuals sampled, as well as the proportion of samples that are cases versus controls [2, 6, 7, 18]. Factors such as study duration, disease prevalence, and specificity of the symptoms which trigger enrollment into the study may impact sample size and case-to-control ratios. To explore how these sample attributes impact the accuracy with which we can infer a protection function, we simulated TND data from a range of sample sizes and case-to-control ratios using a sigmoidal protection function, and the simulated data was used to estimate the protection function from both the scaled logit and logistic regression models. For each scenario, we computed error as the discrete $\ell_2$ norm of the difference between the best-fit model $\hat{\Phi}(A)$ and the true protection function $\Phi_{\text{true}}(A)$, given by

$$\|\hat{\Phi} - \Phi_{\text{true}}\|_{\ell_2} = \left( \sum_{i=1}^{N} \left[ \hat{\Phi}(A_i) - \Phi_{\text{true}}(A_i) \right]^2 \right)^{\frac{1}{2}}, \tag{S3}$$

where $\{A_i\}_{i=1}^{N}$ is the set of all observed antibody titers in the study. To account for stochasticity in the generation of synthetic data, the mean error was calculated for 50 simulations per scenario.

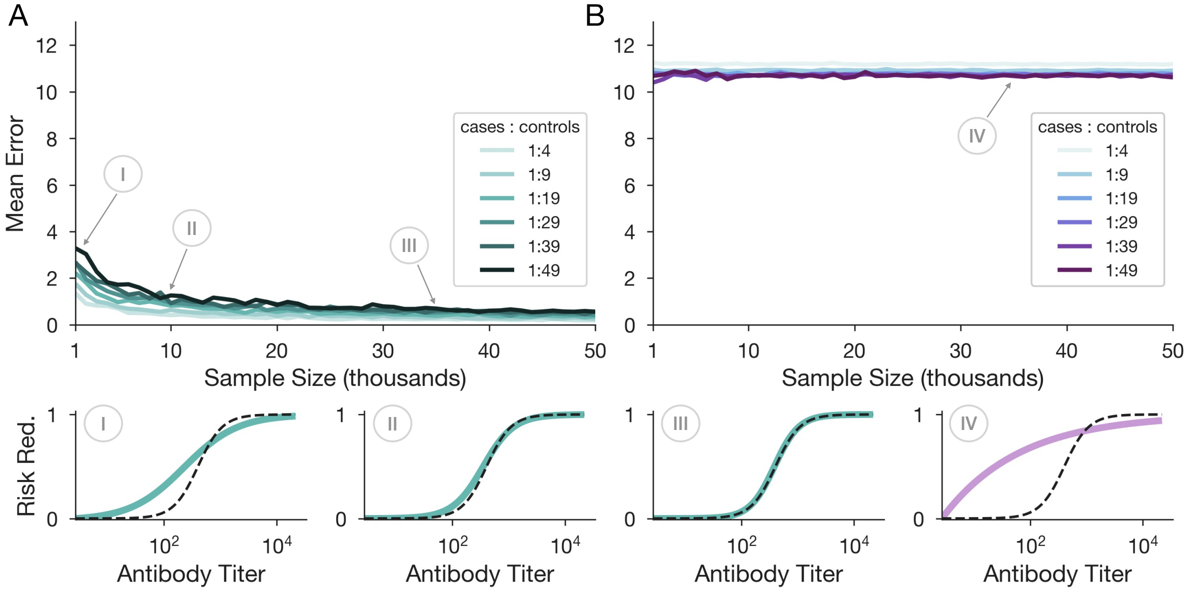


Figure S4: **The scaled logit model is better specified to recover sigmoidal protection from TND data than logistic regression, irrespective of sample size.** Mean error over 50 simulations per scenario for various sample sizes and case-to-control ratios using the scaled logit model (A) and logistic regression (B). Error was defined as the discrete $\ell_2$ norm (Euclidean distance) between the modeled and true protection functions. Darker colored lines represent scenarios with more controls per case. Insets show protection function estimates (colored curve) compared with the true protection function (black dashed curve) from a single simulation when $N = 1$, 10, and 35 thousand for the scaled logit (I-III) and 35 thousand for the logistic model (IV) using a $1 : 49$ case-to-control ratio.

When the scaled logit model is used to estimate protection, we observe higher error for smaller sample sizes (Fig. S4A) due to model overfitting. At low sample sizes, the model is unable to capture the true slope of the sigmoid (panel I). As sample size increases, model fits more closely resemble the true protection

function on average (panels II, III). Furthermore, we observe lower error when a higher proportion of total samples are cases (lighter colored curves), especially at low sample sizes, especially at sample sizes below 1000 (Figs. S5, S6). This demonstrates that cases typically provide more information to model inference than controls.

The same analysis of error in protection function estimation using logistic regression shows uniformly high error across all scenarios (Fig. S4B), which does not approach 0 as sample size increases. These results reflect the large discrepancy between the inferred and true protection functions when using logistic regression to estimate a sigmoidal protection function from TND data (panel IV). This observation offers further support that logistic regression is poorly suited to recover a sigmoidal protection function, at any sample size, due to model misspecification.

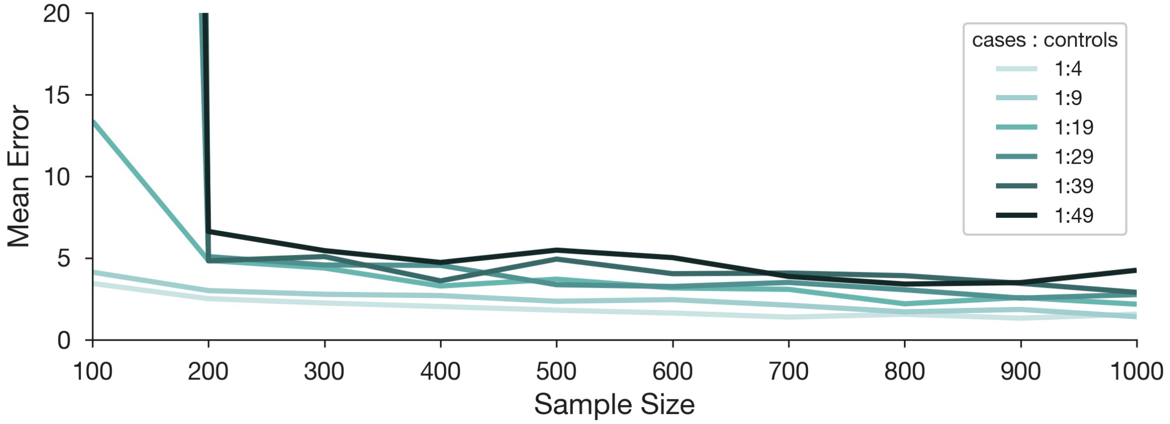


Figure S5: **The scaled logit model has low accuracy at small sample sizes.** Average error over 50 simulations per scenario for various sample sizes and case-to-control ratios using the scaled logit model. Error was defined as the discrete $\ell_2$ norm (Euclidean distance) between the modeled and true protection functions. Darker-colored lines represent scenarios with more controls per case.

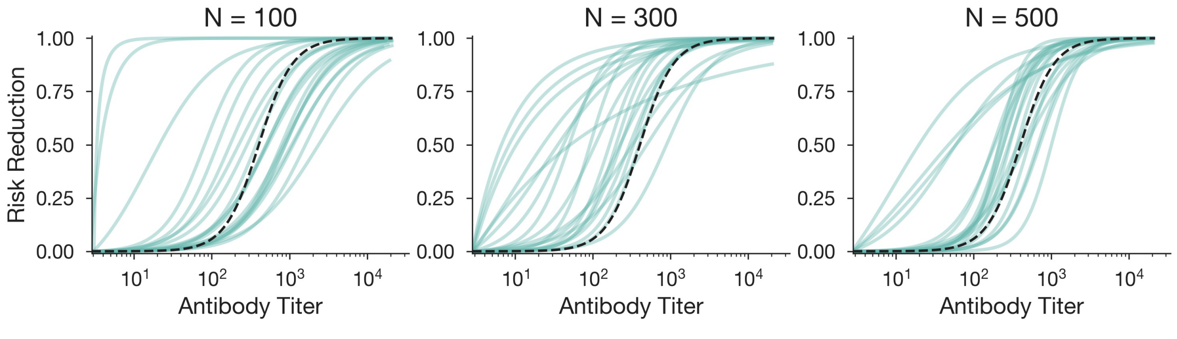


Figure S6: **Scaled logit model fit with low sample sizes.** Estimated protection function using the scaled logit model (green curves) for 50 stochastic simulations at the specified sample size ($N$) using a sigmoidal true protection (dashed black curve).

## Modeling the impact of vaccination and prior infection on immunity

Our primary analysis focused on a single antigen-specific antibody measurement as a correlate of protection. This synthetic data testbed may represent the protective effects of a single protein vaccination, such as SARS-CoV-2 mRNA vaccines, which elicits a protective immune response to only one antigen. However, empirical data may reflect protection from multiple immunological sources if, for example, some individuals have a more diverse immune memory due to prior infection. In this case, unobserved covariates (e.g. prior infection status, unmeasured antigen-specific antibodies, other immune responders) may provide additional protection from disease, making titer a collider variable.

To study how these covariates impact model performance, we developed a secondary synthetic data testbed which includes protection from both vaccination (via measures antibodies, $A$) and prior infection. We assume that vaccination and prior infection are independently distributed with probability 0.7. Individuals with immunity from either vaccination or prior infection alone have $\log$ antibodies drawn from the distribution $A \sim \text{logUniform}(1, 10)$. Individual with hybrid immunity, i.e. both from vaccination and prior infection, are assumed to have a higher titer value of $A_{\text{hybrid}} \sim \text{logUniform}(4, 10)$. Lastly, we assume that those with prior infection receive a 40% boost to immunity, such that the observed infection probability for those with prior infection is $(1 - \Phi(A)) \cdot (1 - 0.4)$. Data is observed for individual $\log$ antibodies, vaccination status, and infection status with perfect sensitivity and specificity.

## Fitting the scaled logit model to data

We use the built in `scipy.minimize` function in python, implemented with the Nelder-Mead minimization algorithm [39] and options to increase the number of iterations before convergence. In python, this process uses the following code, where $\lambda$ has been replaced with $k$ to avoid conflict with built-in functions:

```python
# Import required packages
import numpy as np
from scipy.optimize import minimize

# Define the scaled logit function
def scaled_logit(x, k, beta_0, beta_1):
    """
    Scaled logit function
    Parameters:
        x (array-like): Input values.
        k (float): Maximum value (scale).
        beta_0 (float): Intercept parameter for linear regression.
        beta_1 (float): Slope parameter.

    Returns:
    array-like: Scaled logistic function values.
    """
    return k / (1 + np.exp(beta_0 + beta_1*x))

def neg_log_likelihood_scaled_logit(params, data, lambda_reg = 0.1):
    '''
    Defines negative log-likelihood function for scaled logit model
    Parameters:
        params: list of model params [k, beta_0, beta_1]
        data: list of lists containing [Ab_titers, infected_status] for all oberved samples

    Returns:
        Scalar negative log-likelihood value
    '''
    k, beta_0, beta_1 = params
    Abs = np.array(data[0]); infected = np.array(data[1])

    # Get likelihood
    prob_pos = infected * np.log(scaled_logit(Abs, k, beta_0, beta_1))
    prob_neg = (1-infected) * np.log(1-scaled_logit(Abs, k, beta_0, beta_1))

    # Add L2 regularization penalty on beta_1 to discourage steep slopes
    regularization_penalty = lambda_reg * beta_1**2

    return -1*np.sum(prob_pos + prob_neg) + regularization_penalty
```

```python
def fit_scaled_logit(x_data, y_data, initial_guess=(0.5, 1, -1)):
    """
    Fit the scaled logit model to data.
    Parameters:
        x_data (array-like): Independent variable values.
        y_data (array-like): Dependent variable values.
        initial_guess (tuple): Initial guesses for k, beta_0, and beta_1.

    Returns:
        tuple: Fitted parameters (k, beta_0, beta_1)
    """
    data = [x_data,y_data]
    result = minimize(neg_log_likelihood_scaled_logit,
    initial_guess, method='Nelder-Mead', args=(data, lambda_reg),
    options={'xatol': 1e-10, 'fatol': 1e-10, 'maxiter': 10000, 'maxfev': 20000}
    )
    return result.x

# Example of fitting the model to data

# Generate anibody titers for 1000 cases and controls
Ab_titers_cases = np.random.uniform(np.log(1), np.log(1000), 1000)
Ab_titers_controls = np.random.uniform(np.log(1), np.log(8000), 1000)
Ab_titers = np.concatenate([Ab_titers_cases, Ab_titers_controls])

# Generate infected status array
infected = np.concatenate([np.ones(1000),np.zeros(1000)])

# Fit sccaled logit model to data
fitted_params = fit_scaled_logit(Ab_titers,infected)
fitted_k, fitted_beta_0, fitted_beta_1 = fitted_params
print(fitted_params)

# Create vector of model predictions for increasing titers
potential_Abs = np.arange(np.log(1),np.log(8000))
prob_infection = scaled_logit(potential_Abs, fitted_k, fitted_beta_0, fitted_beta_1)
```

If the researcher prefers to work in R, the equivalent script is:

```r
# Load necessary library
library(stats)

# Define the scaled logit function
scaled_logit <- function(x, k, beta_0, beta_1) {
  return(k / (1 + exp(beta_0 + beta_1 * x)))
}

# Define the negative log-likelihood function for the scaled logit model
neg_log_likelihood_scaled_logit <- function(params, data,lambda_red=0.1) {
  k <- params[1]
  beta_0 <- params[2]
  beta_1 <- params[3]

  Abs <- data[[1]]
  infected <- data[[2]]

  # Compute likelihood
  prob_pos <- infected * log(scaled_logit(Abs, k, beta_0, beta_1))
  prob_neg <- (1 - infected) * log(1 - scaled_logit(Abs, k, beta_0, beta_1))

  # Add L2 regularization penalty on beta_1 to discourage steep slopes
  regularization_penalty <- lambda_reg * beta_1^2

  return(-sum(prob_pos + prob_neg) + regularization_penalty)
}

# Function to fit the scaled logit model
fit_scaled_logit <- function(x_data, y_data, initial_guess = c(0.5, 1, -1)) {
  data <- list(x_data, y_data)

  result <- optim(
    par = initial_guess,
    fn = neg_log_likelihood_scaled_logit,
    data = data,
    method = "Nelder-Mead",
    control = list(reltol = 1e-6, maxit = 4000)
  )

  return(result$par)
}

# Example of fitting the model to data

# Generate antibody titers for 1000 cases and controls
Ab_titers_cases <- runif(1000, log(1), log(1000))
Ab_titers_controls <- runif(1000, log(1), log(8000))
```

```
Ab_titers <- c(Ab_titers_cases, Ab_titers_controls)

# Generate infected status array
infected <- c(rep(1, 1000), rep(0, 1000))

# Fit scaled logit model to data
fitted_params <- fit_scaled_logit(Ab_titers, infected)
fitted_k <- fitted_params[1]
fitted_beta_0 <- fitted_params[2]
fitted_beta_1 <- fitted_params[3]
print(fitted_params)

# Create vector of model predictions for increasing titers
potential_Abs <- seq(log(1), log(8000), length.out = 100)
prob_infection <- scaled_logit(potential_Abs, fitted_k, fitted_beta_0, fitted_beta_1)
```
